# Synergistic Anti-Inflammatory Activity of the Antimicrobial Peptides Human Beta-Defensin-3 (hBD-3) and Cathelicidin (LL-37) in a Three-Dimensional Co-Culture Model of Gingival Epithelial Cells and Fibroblasts

**DOI:** 10.1371/journal.pone.0106766

**Published:** 2014-09-04

**Authors:** Telma Blanca Lombardo Bedran, Márcia Pinto Alves Mayer, Denise Palomari Spolidorio, Daniel Grenier

**Affiliations:** 1 Department of Oral Diagnosis and Surgery, Araraquara Dental School, State University of São Paulo, São Paulo, Brazil; 2 Department of Microbiology, Institute of Biomedical Sciences, University of São Paulo, São Paulo, Brazil; 3 Department of Physiology and Pathology, Araraquara Dental School, State University of São Paulo, São Paulo, Brazil; 4 Oral Ecology Research Group, Faculty of Dentistry, Université Laval, Quebec City, QC, Canada; University Hospital Schleswig-Holstein, Campus Kiel, Germany

## Abstract

Given the spread of antibiotic resistance in bacterial pathogens, antimicrobial peptides that can also modulate the immune response may be a novel approach for effectively controlling periodontal infections. In the present study, we used a three-dimensional (3D) co-culture model of gingival epithelial cells and fibroblasts stimulated with *Aggregatibacter actinomycetemcomitans* lipopolysaccharide (LPS) to investigate the anti-inflammatory properties of human beta-defensin-3 (hBD-3) and cathelicidin (LL-37) and to determine whether these antimicrobial peptides can act in synergy. The 3D co-culture model composed of gingival fibroblasts embedded in a collagen matrix overlaid with gingival epithelial cells had a synergistic effect with respect to the secretion of IL-6 and IL-8 in response to LPS stimulation compared to fibroblasts and epithelial cells alone. The 3D co-culture model was stimulated with non-cytotoxic concentrations of hBD-3 (10 and 20 µM) and LL-37 (0.1 and 0.2 µM) individually and in combination in the presence of *A. actinomycetemcomitans* LPS. A multiplex ELISA assay was used to quantify the secretion of 41 different cytokines. hBD-3 and LL-37 acted in synergy to reduce the secretion of GRO-alpha, G-CSF, IP-10, IL-6, and MCP-1, but only had an additive effect on reducing the secretion of IL-8 in response to *A. actinomycetemcomitans* LPS stimulation. The present study showed that hBD-3 acted in synergy with LL-37 to reduce the secretion of cytokines by an LPS-stimulated 3D model of gingival mucosa. This combination of antimicrobial peptides thus shows promising potential as an adjunctive therapy for treating inflammatory periodontitis.

## Introduction

Periodontitis is a multifactorial chronic inflammatory disease of polymicrobial origin that causes the destruction of the tooth-supporting tissues, including the periodontal ligament and alveolar bone [1]. A limited number of Gram-negative, mostly anaerobic bacteria that colonize the subgingival sites and activate the host immune response have been associated with this disease [2]. More specifically, *Aggregatibacter actinomycetemcomitans* is considered to be a key etiological agent of aggressive periodontitis [3]. The lipopolysaccharide (LPS) of *A. actinomycetemcomitans* is a major virulence factor that can promote adhesion to oral cells and can activate the host immune response, resulting in the secretion of large amounts of pro-inflammatory cytokines, including interleukin-6 (IL-6) and interleukin-8 (IL-8) that contribute to the destruction of periodontal tissues [4]–[6].

Epithelial cells and fibroblasts are the predominant cells of periodontal tissues and serve as a first line of defense against periodontopathogens. They act as a mechanical barrier against bacterial invasion in addition to secreting different classes of inflammatory mediators and tissue-destructive enzymes in response to pathogen stimulation. When the immune and inflammatory responses do not stop the progression of the periodontal infection, uncontrolled secretion of cytokines occurs, leading to chronic inflammation and periodontal tissue destruction [7]. For instance, higher levels of IL-8 and monocyte chemo-attractant protein 1 (MCP-1) have been found in gingival crevicular fluid (GCF) from periodontitis sites than in GCF from healthy control sites, while their levels decrease after periodontal treatments [8]–[12].

Traditional scaling and root planing remain the “gold standard” for the treatment of periodontitis. However, some patients do not respond adequately to this conventional therapy and require adjunctive antimicrobials. Given that many bacteria have developed resistance to antibiotics, new strategies need to be developed for adjunctive therapies [13]. Antimicrobial peptides (AMPs) are small cationic molecules of the innate immune response with a broad activity spectrum against pathogens, including those associated with periodontitis [14]. Gingival epithelial cells have been reported to secrete several AMPs either constitutively or in response to an infection [14], [15]. LL-37 and human β-defensin (hBD-3) are the most important AMPs found in humans. CAP18 is the only member of the cathelicidin family found in humans. The C-terminal of CAP18 is proteolytically cleaved to generate LL-37, a 37-amino-acid peptide beginning with two leucine residues [16],[17]. hBD-3 is an important defensin in the oral cavity and is expressed in response to bacterial invasion [15], [18]. Both hBD-3 and LL-37 have a broad activity spectrum and have been detected in GCF and saliva [19]–[22]. Some studies have reported a marked reduction in the amounts of hBD-3 and LL-37 in gingival crevicular fluid during periodontitis, which could be related to the ability of certain periodontopathogens to proteolytically inactivate the peptides or down-regulate their expression [23]–[25].

hBD-3 and LL-37 show antimicrobial activity against a broad range of oral Gram-positive and Gram-negative bacteria [26], [27]. These positively charged cationic peptides bind to the negatively charged bacterial membrane components leading to the formation of pores and cell lysis [28]–[30]. In addition to exert antimicrobial activity, both peptides modulate the immune response [31]. hBD-3 and LL-37 can neutralize the inflammatory potential of LPS by binding directly to LPS or by preventing the binding of LPS to host cell receptors, thus blocking the cell signaling pathway triggered by TLR ligands [32]–[34]. Walters et al. [35] showed that LL-37 reduces cytokine secretion by LPS-stimulated human whole blood and proposed that this peptide should be considered for use in adjunctive periodontal treatments. By acting on the two etiological components of periodontitis, i.e., periodontopathogens and the inflammatory response, hBD-3 and LL-37 are very attractive candidates for adjunctive periodontal treatments.

Previous studies have shown that hBD-3 and LL-37 may work in association with other antimicrobial agents to enhance their antibacterial activities [36]–[38]. More specifically, Chen et al. [39] reported that hBD-3 and LL-37 work in synergy to inhibit the growth of *Staphylococcus aureus.* This may be related to the fact that hBD-3 and LL-37 are able to act on different cell targets. In this study, we hypothesized that synergistic interactions between hBD-3 and LL-37 may also exist in regard to reduction of LPS-induced inflammatory response of host cells. Consequently, we used an in vitro three-dimensional (3D) co-culture model of gingival epithelial cells and fibroblasts stimulated with *A. actinomycetemcomitans* LPS to determine the effect of cell interactions on cytokine secretion and to investigate the synergistic anti-inflammatory activities of hBD-3 and LL-37.

## Materials and Methods

### Antimicrobial peptides and LPS preparation

The synthetic hBD-3 (H-GIINTLQKYYCRVRGGRCAVLSCLPKEEQIGKCSTRGRKCCRRKK-OH) and LL-37 (H-LLGDFFRKSKEKIGKEFKRIVQRIKDFLRNLVPRTES-OH) peptides were from Biomatik (Cambridge, ON, Canada). They were dissolved in sterile UltraPure DNase/RNase-free distilled water (Life Technologies Inc., Burlington, ON, Canada) at a concentration of 1 mM and were stored at −20°C until used. *A. actinomycetemcomitans* (ATCC 29522) LPS was isolated using the protocol previously described by Darveau and Hancock [40]. Stock solutions (1 mg/mL) prepared in sterile distilled water were stored at −20°C until used.

### Cultivation of gingival epithelial cells and fibroblasts

The immortalized human gingival epithelial cell line OBA-9 [41], which was kindly provided by M. Mayer (Department of Microbiology, Institute of Biomedical Sciences, University of São Paulo, São Paulo, Brazil), was cultured in keratinocyte serum-free medium (K-SFM; Life Technologies Inc.) containing insulin, epidermal growth factor, fibroblast growth factor, and 100 µg/mL of penicillin G-streptomycin. The primary human gingival fibroblast cell line HGF-1 (ATCC CRL-2014) was purchased from the American Type Culture Collection (ATCC) (Manassas, VA, USA) and was cultured in Dulbecco’s modified Eagle’s medium (DMEM) supplemented with 4 mM L-glutamine (HyClone Laboratories, Logan, UT, USA), 10% heat-inactivated fetal bovine serum (FBS), and 100 µg/mL of penicillin G-streptomycin. Both cell lines were incubated at 37°C in a 5% CO_2_ atmosphere until they reached confluence.

### Preparation of the three-dimensional (3D) co-culture model

**Figure 1:**
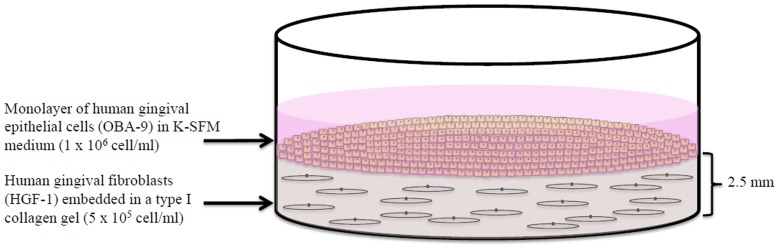
Schematic representation of the 3D co-culture model. The model is composed of gingival fibroblasts (HGF-1) embedded in a collagen matrix overlaid with gingival epithelial cells (OBA-9) and is a modification of the model described by Gursoy et al. [42].

A 3D co-culture model composed of gingival fibroblasts embedded in a collagen matrix overlaid with gingival epithelial cells was prepared according to the protocol described by Gursoy et al. [42], with slight modifications. Preliminary assays allowed to determine the incubation times to be used to obtain fibroblast and epithelial cell differentiation with a cell confluence of approximately 80%. A commercial bovine type I collagen solution (95–98%; PureCol, Advanced BioMatrix, Tucson, AZ, USA) was mixed with DMEM (10X) (Sigma-Aldrich Canada, Oakville, ON, Canada) on ice to obtain a final collagen concentration of 76–78%. The pH was adjusted to 7. Confluent HGF-1 cells were detached by gentle trypsinization (0.05% trypsin-EDTA; Gibco-BRL, Grand Island, NY, USA). The trypsin was then inactivated by adding DMEM +10% FBS. The cells were harvested by centrifugation (500×g for 5 min) and were suspended at a density of 5×10^5^ cells/mL in the collagen solution described above. The collagen cell suspension was placed in the wells of 6-well tissue culture plates (2 mL/well; 2.5-mm-thick) (Sarstedt, Newton, NC, USA). The collagen gel was allowed to solidify for 2 h at 37°C under aerobic conditions, and the plates were then incubated for a further 10 h at 37°C in a 5% CO_2_ atmosphere. The OBA-9 cells were detached by gentle trypsinization (5 min) (TrypLE Express; Life Technologies Inc.) at 37°C. The trypsin was then inactivated by adding 0.3 mg/mL of trypsin inhibitor, and the cells were harvested by centrifugation (500×g for 5 min) and suspended in fresh K-SFM medium. Aliquots (2 mL) of OBA-9 cells were seeded on top of the collagen-fibroblast gels at a density of 1×10^6^ cell/mL. The 3D co-culture model (Figure 1) was incubated overnight at 37°C in a 5% CO_2_ atmosphere to allow cell adhesion prior to stimulation.

### Comparative analysis of LPS-induced IL-6 and IL-8 secretion by the 3D co-culture model and the individual cell lines

The 3D co-culture model was stimulated with *A. actinomycetemcomitans* LPS (1 µg/mL) for 24 h at 37°C in a 5% CO_2_ atmosphere. Unstimulated cells (individually and in co-culture) were used as controls. The supernatants were collected, centrifuged (3000×g for 10 min at 4°C), and then stored at −20°C until used for the IL-6 and IL-8 assays. After a 24-h incubation, the co-culture model, the collagen-fibroblast gel, and the epithelial cells were visualized by inverted phase-light microscopy. Commercial enzyme-linked immunosorbent assay (ELISA) kits (eBioscence, Inc., San Diego, CA, USA) were used to quantify the IL-6 and IL-8 concentrations in the culture supernatants according to the manufacturer’s protocols. The absorbance at 450 nm (A_450_) was recorded using a microplate reader with the wavelength correction set at 570 nm. Assays were performed in triplicate in two independent experiments and the means ± standard deviations were calculated.

### Stimulation of the 3D co-culture model

The 3D co-culture model was pre-treated for 2 h with hBD-3 (10 and 20 µM) or LL-37 (0.1 and 0.2 µM), or both, prior to being stimulated with *A. actinomycetemcomitans* LPS (1 µg/mL) for 24 h at 37°C in a 5% CO_2_ atmosphere. These concentrations of hBD-3 and LL-37 were selected based on preliminary assays in the 3D co-culture model that showed that such amounts of antimicrobial peptides were not cytotoxic and provided a moderate anti-inflammatory effect (data not shown). Co-cultures not pre-treated with hBD-3 or LL-37 and not stimulated with LPS were used as controls. The supernatants were collected, centrifuged (1000×g for 5 min at 4°C), and stored at −20°C until used. Assays were performed in triplicate.

### Determination of the viability of the OBA-9 and HGF-1 cell lines

We determined the effect of hBD-3, LL-37, and *A. actinomycetemcomitans* LPS, individually and in combination, on the viability of the OBA-9 and HGF-1 cells. Briefly, HGF-1 and OBA-9 cells (1×10^4^ cells/well) were seeded in the wells of a 96-well microplate (0.1 mL/well) (Sarstedt) and were incubated for 4 h at 37°C in a 5% CO_2_ atmosphere to allow cell adhesion. The culture medium was then aspirated, and the cells were pre-treated for 2 h with hBD-3 (5, 10, 20, 40 µM) and/or LL-37 (0.05, 0.1, 0.2, 0.5, 1, 5 µM) prior to adding 1 µg/mL of *A. actinomycetemcomitans* LPS. The cells were incubated for an additional 24 h at 37°C in a 5% CO_2_ atmosphere. A colorimetric MTT cell viability assay (Roche Diagnostics, Mannheim, Germany) using 3-[4,5-diethylthiazol-2-yl]-2,5-diphenyltetrazolium bromide as the substrate was performed according to the manufacturer’s protocol. Untreated control cells were assigned a value of 100%, and all the other conditions were compared to the control. Results are expressed as means ± standard deviations of duplicate assays from two independent experiments.

### Determination of cytokine secretion using multiplex ELISA assays

Samples of the 3D co-culture model subjected to the various treatments were sent to Eve Technologies (Calgary, AB, Canada, http://www.evetechnologies.com) for multiplex ELISA analyses. Eve Technologies uses the Bio-Plex Suspension Array System to quantify 41 different cytokines, chemokines, and growth factors (Human 41-Plex Discovery Assay): epidermal growth factor (EGF), C-C motif chemokine 11 (Eotaxin-1), basic fibroblast growth factor (FGF-2), FMS-like tyrosine kinase 3 ligand (Flt3l), chemokine (C-X3-C motif) ligand 1 (Fractalkine), granulocyte colony-stimulating factor (G-CSF), granulocyte-macrophage colony-stimulating factor (GM-CSF), CXC-chemokine ligand 1 (GRO-α), interferon alpha 2 (IFN-α2), interferon gamma (IFNγ), interleukin-1 alpha (IL-1α), interleukin-1 beta (IL-1β), interleukin-1 receptor antagonist (IL-1ra), interleukin-2 (IL-2), interleukin-3 (IL-3), interleukin-4 (IL-4), interleukin-5 (IL-5), interleukin-6 (IL-6), interleukin-7 (IL-7), interleukin-8 (IL-8), interleukin-9 (IL-9), interleukin-10 (IL-10), interleukin-12B (IL-12B), interleukin-12 (p70) [IL-12 (p70)], interleukin-13 (IL-13), interleukin-15 (IL-15), interleukin 17A (IL-17A), interferon-γ inducible protein 10 (IP-10), monocyte chemo-attractant protein 1 (MCP-1), monocyte-specific chemokine 3 (MCP-3), C-C motif chemokine 22 (MDC), macrophage inflammatory protein 1α (MIP-1α), macrophage inflammatory protein 1β (MIP-1β), platelet-derived growth factor AA (PDGF-AA), platelet-derived growth factor AB/BB (PDGF-AB/BB), regulated on activation, normal T cell expressed and secreted (RANTES), soluble CD40 ligand (sCD40L), transforming growth factor alpha (TGF-α), tumor necrosis factor alpha (TNF-α), tumor necrosis factor beta (TNF-β), and vascular endothelial growth factor A (VEGF-A).

### Data analysis

To determine the synergistic inhibitory effect of hBD-3 and LL-37 on cytokine secretion following stimulation of the 3D co-culture model with *A. actinomycetemcomitans* LPS, the sums of the inhibition values of each peptide were compared with the values of both compounds used in combination. Experiments were carried out a minimum of three times to ensure reproducibility. The means ± SD from a representative experiment are presented. Differences between the means were analyzed for statistical significance using a one-way ANOVA. Statistical significance was set at p<0.05.

## Results

**Figure 2:**
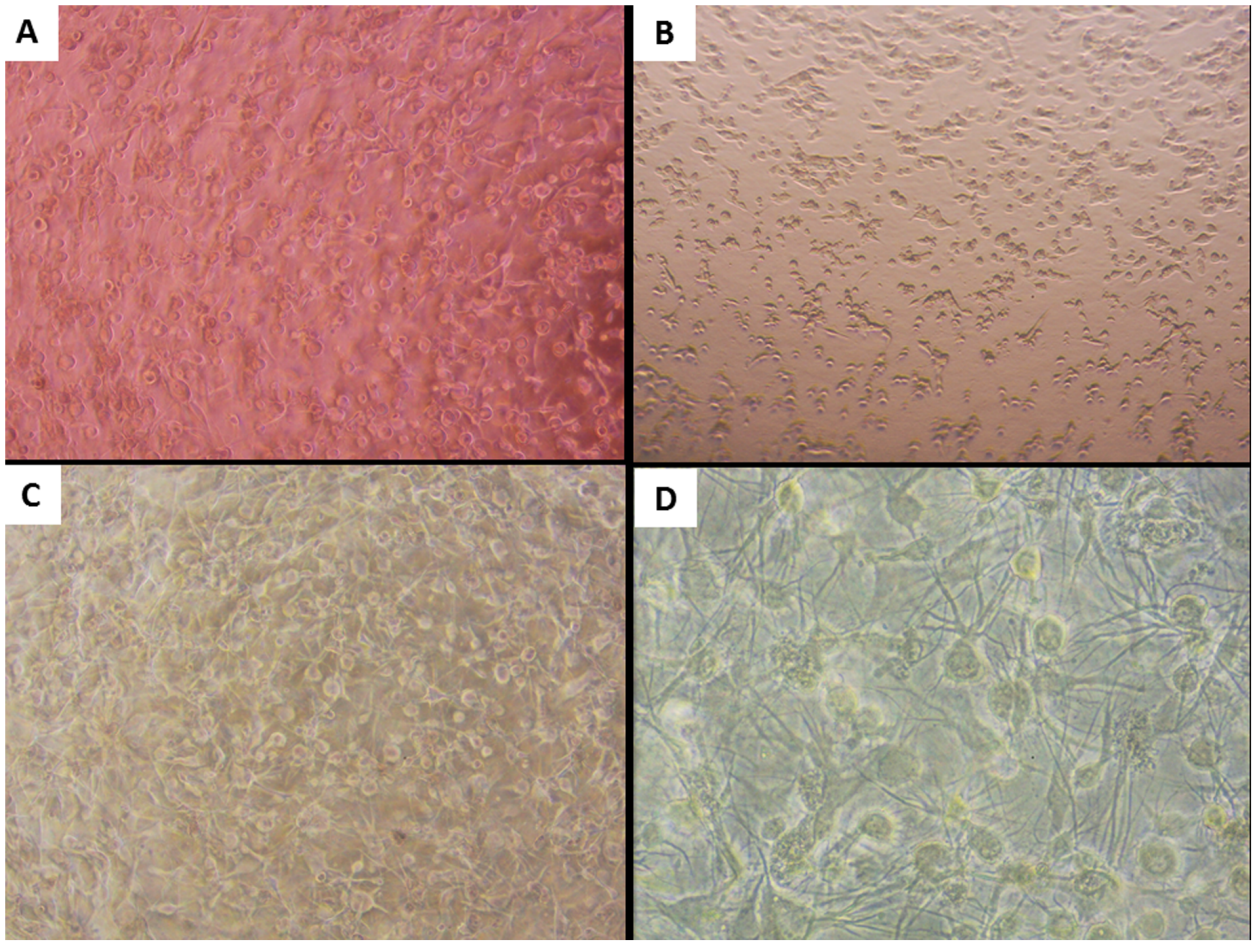
Light microscopic observations of the individual cell lines and the 3D co-culture model. A: Collagen-gingival fibroblast (HGF-1) gel; B: Gingival epithelial cells (OBA-9) seeded on the collagen gel; C and D: 3D co-culture model composed of gingival fibroblasts embedded in a collagen matrix overlaid with gingival epithelial cells. The epithelial cells, fibroblasts, and 3D co-culture model were stimulated with *A. actinomycetemcomitans* LPS (1 µg/mL) for 24 h at 37°C in a 5% CO_2_ atmosphere. Panels A, B, and C (4× magnification). Panel D (10× magnification).

The HGF-1 gingival fibroblast cells (Figure 2A), OBA-9 gingival epithelial cells (Figure 2B), and the 3D co-culture model composed of both cell types were observed by light microscopy. Given the density and close proximity of epithelial cells and fibroblasts in the 3D co-culture model, several interactions between the two cells types are likely to occur.

**Figure 3:**
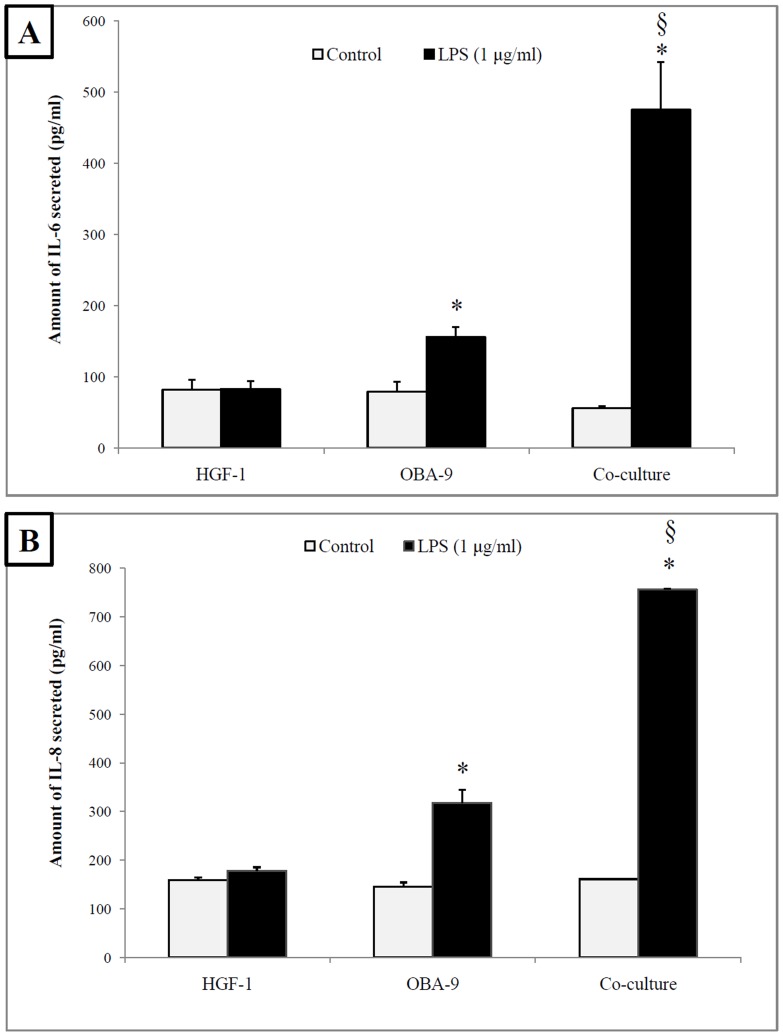
Amount of IL-6 (A) and IL-8 (B) secreted by gingival fibroblasts (HGF-1), gingival epithelial cells (OBA-9), and the 3D co-culture model in the absence and presence of *A. actinomycetemcomitans* LPS (1 µg/mL). Results are expressed as means ± standard deviation of triplicate assays from two independent experiments. *, *p*<0.05: significantly different from the control cells without LPS stimulation for the individual cell lines and the 3D co-culture model; §, *p*<0.05: synergistic effect of the cells in the 3D co-culture model compared to the individual cell lines.

Preliminary experiments showed that the treatment of the gingival fibroblasts and epithelial cells with 1 µg/mL of *A. actinomycetemcomitans* LPS had no cytotoxic effect (data not shown). To determine whether the interactions between the two cell types modified the response to LPS stimulation, the secretion of IL-6 and IL-8 by each cell type and by the 3D co-culture model was determined by ELISA. While the stimulation of the fibroblasts with LPS did not significantly increase IL-6 and IL-8 secretion, the stimulation of the epithelial cells with LPS resulted in the secretion of higher amounts of both cytokines compared to the unstimulated cells (Figure 3). More specifically, the secretion of IL-6 and IL-8 by epithelial cells increased by 97% and 120%, respectively, in the presence of LPS. Interestingly, the LPS-stimulated 3D co-culture model had a synergistic response with respect to the secretion of IL-6 and IL-8 compared to that of the individual cell types (Figure 3), secreting 475 pg/mL of IL-6 and 756 pg/mL of IL-8 compared to 239 pg/mL and 496 pg/mL, respectively, by the individual cell lines. No synergistic effect was observed in the absence of LPS stimulation (Figure 3).

**Figure 4:**
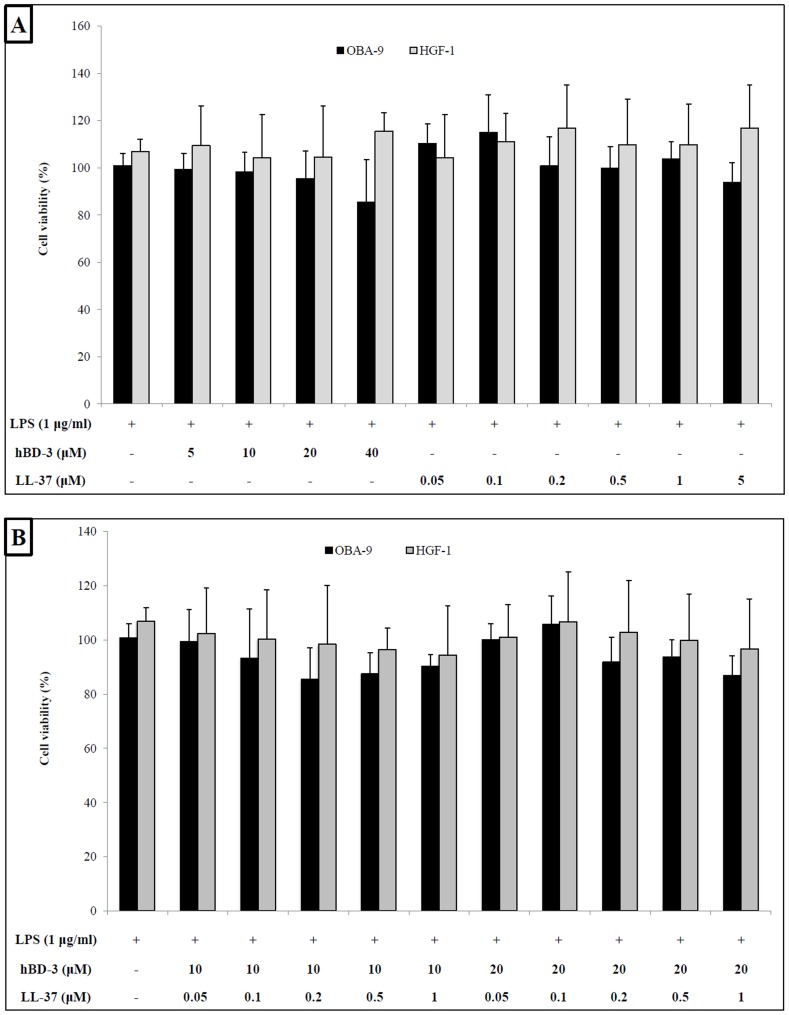
Effect of hBD-3 and LL-37 alone (A) and in combination (B) on the viability of LPS-stimulated gingival fibroblasts (HGF-1) and gingival epithelial cells (OBA-9). Untreated cells were assigned a value of 100%. All the other stimulations were compared to the control. Results are expressed as means ± standard deviation of duplicate assays from two independent experiments. No statistical significance was observed using ANOVA.

Prior to investigating the anti-inflammatory potential of hBD-3 and LL-37 in the 3D co-culture model, we determined their effect on the viability of LPS-stimulated gingival epithelial cells and gingival fibroblasts. None of the concentrations of hBD-3 (5, 10, 20, and 40 µM) and LL-37 (0.05, 0.1, 0.2, 0.5, 1, and 5 µM) tested had a significant effect on the viability of either cell type (Figure 4A). We then determined the effect of combinations of concentrations of hBD-3 (10 and 20 µM) and LL-37 (0.05, 0.1, 0.2, 0.5, and 1 µM) on cell viability. None of the combinations of hBD-3 and LL-37 had a cytotoxic effect on the OBA-9 and HGF-1 cells (Figure 4B). The viability of the 3D co-culture model was also not affected by hBD-3 and LL-37 (data not shown).

**Figure 5:**
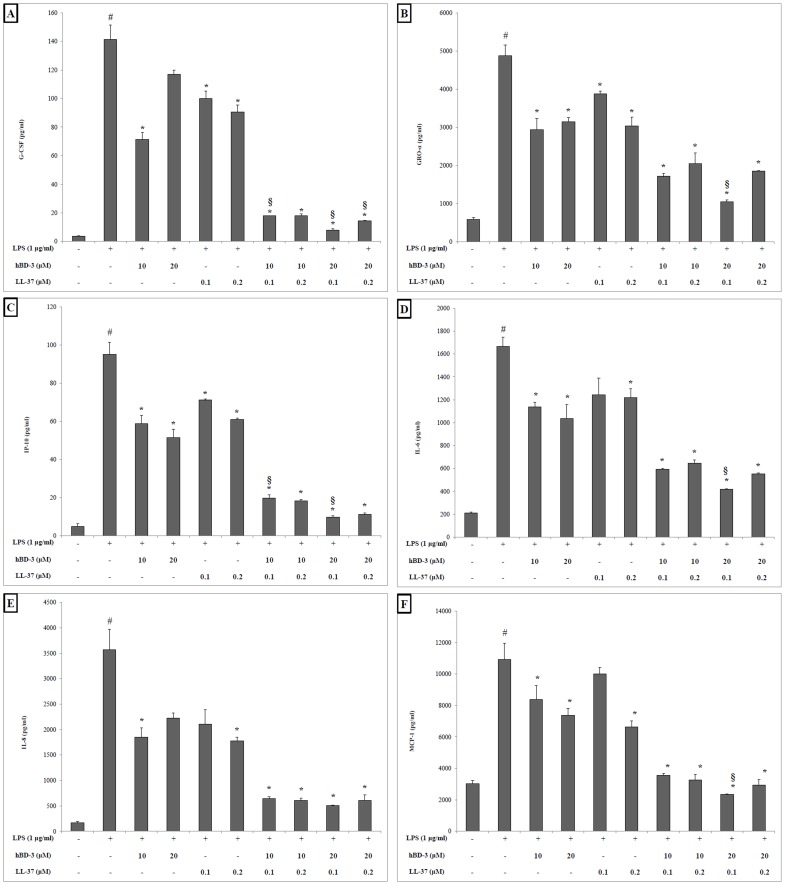
Effect of hBD-3 and LL-37 alone and in combination on the secretion of G-CFS (A), GRO-α (B), IP-10 (C), IL-6 (D), IL-8 (E), and MCP-1 (F) by the LPS-stimulated 3D co-culture model. Results are expressed as means ± standard deviation of triplicate assays from two independent experiments. ^#^, significantly higher than the unstimulated (LPS) negative control (*p*<0.01); *, significantly lower than the untreated (hBD-3, LL-37) positive control (*p*<0.05); §, synergistic effect of the peptides; significantly lower than the sum of the inhibitory values of each peptide alone (*p*<0.05) compared to the two peptides in combination.

The LPS-stimulated 3D co-culture model was then used to investigate the anti-inflammatory properties of hBD-3 (10 and 20 µM) and LL-37 (0.1 and 0.2 µM) alone and in combination. The concentrations of 41 cytokines, chemokines, and growth factors in the cell-free culture supernatants were assayed using a multiplex ELISA assay. Only G-CSF, GRO-α, IP-10, IL-6, IL-8, and MCP-1 were detected in the culture supernatants. *A. actinomycetemcomitans* LPS significantly increased the secretion of G-CFS (36-fold), GRO-α (8-fold), IP-10 (20-fold), IL-6 (10-fold), IL-8 (20-fold), and MCP-1 (5-fold) by the 3D co-culture model compared to the unstimulated control (Figure 5). In the absence of LPS stimulation, hBD-3 and LL-37 did not induce any secretion of the above factors (data not shown). While the secretion of the cytokines was significantly reduced by 10 and 20 µM hBD-3 and by 0.1 and 0.2 µM LL-37 alone, only the secretion of GRO-α and IP-10 was significantly reduced by all the concentrations of hBD-3 and LL-37 tested following the stimulation of the 3D co-culture model with LPS. hBD-3 (20 µM) and LL-37 (0.1 µM) in combination synergistically inhibited the secretion of five of the six cytokines (G-CSF, GRO-α, IP-10, IL-6, and MCP-1) by the LPS-stimulated 3D co-culture model (Figure 5). All the concentrations of hBD-3 and LL-37 tested in combination had a synergistic inhibitory effect on the secretion of G-CSF. None of the concentrations of hBD-3 and LL-37 tested in combination had a synergistic inhibitory effect on the secretion of IL-8.

## Discussion

Previous studies have shown that hBD-3 and LL-37 display anti-inflammatory activity in monocultures of fibroblasts, monocytes, macrophages, and periodontal ligament cells [43]–[46]. In this study, we investigated for the first time the anti-inflammatory activities of hBD-3 and LL-37, individually and in association, in a 3D co-culture model of gingival epithelial cells and fibroblasts, the two main cell types of the periodontium. Cytokine secretion by the co-culture model was induced by *A. actinomycetemcomitans* LPS based on a previous study showing that among LPS isolated from a number of periodontopathogens, the one of *A. actinomycetemcomitans* induced the highest pro-inflammatory response [47].

The 3D co-culture model is advantageous in that it takes interactions between gingival epithelial cells and fibroblasts into consideration. It has previously been shown that gingival fibroblasts stimulate the proliferation of keratinocytes, while keratinocytes induce the expression of specific fibroblast genes [48], [49]. Gron et al. [50] reported that cultivating oral fibroblasts and keratinocytes together increases the secretion of keratinocyte and hepatocyte growth factors, thus modulating the proliferation and migration of the junctional epithelium. Interactions between gingival fibroblasts and epithelial cells were also observed in our study as evidenced by the fact that the amounts of IL-6 and IL-8 secreted by the LPS-stimulated 3D co-culture model are significantly higher than the amounts secreted by the individual cell lines.

There has been growing interest in recent years in the synergistic antimicrobial and anti-inflammatory properties of various compounds, more particularly because many diseases, including periodontitis, have a multifactorial etiology. During periodontitis, periodontopathogens activate the host inflammatory response, resulting in the secretion of pro-inflammatory mediators, which in turn modulate the destruction of tooth-supporting tissues [7]. Compounds such as AMPs that possess both antimicrobial and anti-inflammatory properties may be potential alternatives to antibiotics in adjunctive therapies for treating periodontitis [51]. In addition to their antimicrobial properties, some AMPs can modulate the immune response and can bind directly to LPS, preventing the binding of LPS to the CD14 receptor and thus inhibiting the secretion of some pro-inflammatory cytokines [32], [52]. Some AMPs can also bind to LPS when it is already bound to macrophage receptors [53]. While a wide variety of human AMPs have been identified, cathelicidins and defensins are the two most thoroughly characterized families. LL-37 is the only member of cathelicidin family present in humans and is produced by several cell types, including epithelial cells, monocytes, and natural killer cells [17], [54], [55]. In addition to have antibacterial activity, it also possesses a broad range of immuno-modulatory effects that allow it to interact with host cell membrane receptors and to inhibit the interaction between these receptors and pathogens [17], [54], [55]. Defensins are found in humans, animals, and plants, and can interact with and disrupt the lipid membranes of microbial pathogens inducing bacterial lysis [29]. More specifically, hBD-3 is expressed by epithelial cells, and has also been shown to possess anti-inflammatory properties [14], [15], [21].

hBD-3 and LL-37 were selected to investigate their anti-inflammatory synergistic effect in the 3D co-culture model for several reasons. First, since these antimicrobial peptides belong to different families, we hypothesized that they are more likely to act in synergy given that hBD-3 modulates the immune response by binding to the TLR4 receptor, that LL-37 can bind to the TLR1/2 and TLR4 receptors [34], [45]. Second, hBD-3 is the predominant defensin in the oral cavity and is produced and stored by cells in the gingival epithelium [56]–[58]. Lastly, many investigators have reported that hBD-3 and LL-37 possess anti-inflammatory properties [43]–[46], although the peptides were tested individually. Pingel et al. [59] reported that hBD-3 can significantly decrease the secretion of IL-6, IL-10, GM-CSF, and TNF-α by human myeloid dendritic cells stimulated with recombinant *Porphyromonas gingivalis* hemagglutinin B (rHagB), while Semple et al. [45] showed that hBD-3 inhibits the secretion of TNF-α and IL-6 by macrophages stimulated with *Escherichia coli* LPS. LL-37 is a potent LPS-neutralizing peptide [52], [53] and strongly suppresses *E. coli* LPS- and *P. gingivalis* LPS-induced IL-6, IL-8, and CXCL 10 secretion by gingival fibroblasts [60]. In addition, Lee et al. [32] recently reported that LL-37 suppresses the pro-inflammatory activities of LPS from *Prevotella intermedia* and *Tannerella forsythia* in both monocytes and gingival fibroblasts.

We investigated the effect of hBD-3 and LL-37, individually and in combination, on cytokine secretion by the 3D co-culture model of gingival epithelial cells and fibroblasts stimulated with A. *actinomycetemcomitans* LPS. This model provides a better interpretation of the inflammatory process since it takes into consideration the possible synergistic effect mediated by cell interactions on cytokine secretion. The 3D co-culture model secreted higher levels of MCP-11, GRO-α, IL-6, and IL-8 and, to a lesser extent, IP-10 and G-CSF in response to *A. actinomycetemcomitans* LPS. All of these molecules may contribute in different ways to the progression of periodontitis. As reported by Sager et al. [61], GRO-α induces an intense inflammatory response when injected into mice, contributing to the degradation of the extracellular matrix and promoting leukocyte infiltration. Kurtis et al. [9] reported that the concentration of MCP-1 in the GCF from diseased sites is significantly higher than in the GCF from healthy sites. Moreover, IL-6 and IL-8 are important inflammatory mediators secreted by macrophages, fibroblasts, and epithelial cells and are found in high concentrations in inflamed gingival and periodontal tissues [62]–[64]. Almasri et al. [65] reported that gingival fibroblasts stimulated with *P. gingivalis* LPS secrete more GRO-α, IL-6, IL-8, and MCP-1 than unstimulated controls, which is in agreement with our results.

Our results showed that 10 µM hBD-3 and 0.2 µM LL-37 alone significantly reduce the secretion of G-CSF, GRO-α, IL-6, IL-8, IP-10, and MCP-1 by the 3D co-culture model in response to LPS. In addition to the anti-inflammatory property of each peptide alone, the combination of 20 µM hBD-3 and 0.1 µM LL-37 synergistically reduced the secretion of GM-SCF, GRO-α, IL-6, IP-10, and MCP-1 in response to LPS. However, the combination of hBD-3 and LL-37 only had an additive effect on reducing the secretion of IL-8 at all the concentrations tested compared to the 3D co-culture model. To the best of our knowledge, no study has reported that a combination of AMPs can exert a synergistic anti-inflammatory effect. However, Semple et al. [45] showed that the association of hBD-3 with 8-bromoadenosine-cAMP (8Br-cAMP), a membrane permeable cAMP analogue, reduces the secretion of TNF-α by mouse macrophages (RAW 264.7) more than hBD-3 or 8Br-cAMP alone. Semple et al. [45] concluded that hBD-3 acts through toll-like receptor 4 (TLR4) while evidence has been brought that LL-37 acts through both TLR4 and TLR1/2 [66]. The combination of the two peptides used in this study may thus be more effective in reducing pro-inflammatory cytokine secretion because they act through different signaling pathways.

It is still unclear whether hBD-3 and LL-37 are pro-inflammatory or anti-inflammatory. hBD-3 may behave like LL-37, which is a multifunctional modulator of the immune response and which is pro-inflammatory at high concentrations and anti-inflammatory activity at lower concentrations [67]. This may explain why hBD-3 at low concentrations (10 µM) was able to reduce the production of G-CSF and IL-8, although at higher concentration (20 µM) hBD-3 was not able to significantly reduce the production of those cytokines (Figure 5).

In summary, the 3D co-culture model used in the present study takes into consideration the interactions that may occur between different cell types (epithelial cells and fibroblasts) and thus mimics the in vivo condition more accurately compared to individual cell types. The 3D co-culture model produced a synergistic increase in the secretion of IL-6 and IL-8 following *A. actinomycetemcomitans* LPS stimulation compared to the individual cell lines. In addition, while hBD-3 and LL-37 both displayed anti-inflammatory activity when applied individually to the model, they acted in synergy when applied together. This suggests that the combination of the two AMPs could be a valuable strategy for replacing antibiotics in adjunctive therapies for the treatment of periodontitis. Further studies are required to investigate the effect of hBD-3 and LL-37 in vivo.
